# Automata Tutor v3

**DOI:** 10.1007/978-3-030-53291-8_1

**Published:** 2020-06-16

**Authors:** Loris D’Antoni, Martin Helfrich, Jan Kretinsky, Emanuel Ramneantu, Maximilian Weininger

**Affiliations:** 8 Microsoft Research Lab, Redmond, WA USA; 9 University of Southern California, Los Angeles, CA USA; 10 University of Wisconsin, Madison, USA; 11 Technical University of Munich, Munich, Germany

**Keywords:** Theory of computation, Automata theory, Personalized education, Automata tutor, Automated grading

## Abstract

Computer science class enrollments have rapidly risen in the past decade. With current class sizes, standard approaches to grading and providing personalized feedback are no longer possible and new techniques become both feasible and necessary. In this paper, we present the third version of Automata Tutor, a tool for helping teachers and students in large courses on automata and formal languages. The second version of Automata Tutor supported automatic grading and feedback for finite-automata constructions and has already been used by thousands of users in dozens of countries. This new version of Automata Tutor supports automated grading and feedback generation for a greatly extended variety of new problems, including problems that ask students to create regular expressions, context-free grammars, pushdown automata and Turing machines corresponding to a given description, and problems about converting between equivalent models - e.g., from regular expressions to nondeterministic finite automata. Moreover, for several problems, this new version also enables teachers and students to automatically generate new problem instances. We also present the results of a survey run on a class of 950 students, which shows very positive results about the usability and usefulness of the tool.

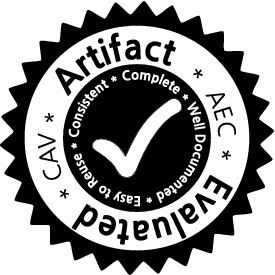



## Introduction

Computer science (CS) class enrollments have been rapidly rising, e.g., CS enrollment roughly triples per decade at Berkeley and Stanford [12] or TU Munich. Both online and offline courses and degrees are being created to educate students and professionals in computer science and these courses may soon have thousands of students attending a lecture, or tens of thousands following a Massive Online Open Course (MOOC). At these scales, standard approaches to grading and providing personalized feedback are no longer possible and new techniques become both feasible and necessary. Current approaches for handling this growing student volume include reducing the complexity of assignments or relying on imprecise feedback and grading mechanisms. Simpler assessment mechanisms, e.g., multiple-choice questions, are easier to grade automatically but lack realism [8]. Designing better techniques for automated grading and feedback generation is therefore a necessity.

Recent advances in formal methods, including program synthesis and verification, can help teachers and students in verifiably correct ways that statistical or rule-based techniques cannot. For example, formal methods have been used to identify student errors and provide feedback for problems related to introductory Python programming assignments [17] geometry [9, 11], algebra [16], logic [2], and automata [3, 6]. In particular, for this last topic, the tool Automata Tutor v2 [7] has already been used by more than 9,000 students at more than 30 universities in North America, South America, Europe, and Asia.

In this paper, we present Automata Tutor v3, an online^1^ tool that extends Automata Tutor v2 and uses techniques from program synthesis and decision procedures to improve the quality and effectiveness of teaching courses on automata and formal languages. Besides being part of the standard CS curriculum, the concepts taught in these courses are rich in structure and applications, e.g., in control theory, text editors, lexical analyzers, or models of software interfaces. Concrete topics in such curricula include automata, regular expressions, context-free grammars, and Turing machines. For problems and assignments related to these topics Automata Tutor v3 can automatically: (1) Detect whether the student’s solution is correct. (2) Detect different types of student’s mistakes and translate them into explanatory feedback. (3) If possible, generate new problems together with the corresponding solutions for teachers to use in class.

Automata Tutor v3 greatly expands its predecessor Automata Tutor v2, which only provides ways to pose and solve problems for deterministic and nondeterministic finite automata constructions. This paper describes the new components introduced by Automata Tutor v3 and how this new version improves on its previous one. The key advantages to its competitors are the breadth, automatic generation and grading of exercises, infrastructure allowing for use in large courses and a useful feedback to the students, compared to text-based interfaces used by Autotool [13], rudimentary feedback in JFLAP [14] and none in Gradience [1].

Since Automata Tutor has already been well received by teachers around the world, we believe that the readers from the CAV community will find great value in knowing about this new and fundamentally richer version of the tool and how it can extensively help with teaching the automata and formal languages courses, a task we know many of the attendees have to face on a yearly basis.

Our contributions are the following:

- **Twelve new types of problems** (added to the four problems from the previous version) that can be created by teachers and for which the *tool can assign grades together with feedback* to student attempts. While the previous version of Automata Tutor could only support problems involving finite automata constructions, Automata Tutor v3 now supports problems for proving language non-regularity using the pumping lemma, building regular expressions, context free grammars, pushdown automata and Turing machines, and conversions between such models.
- **Automatic problem generation** for five types of problems, with the code modularity allowing to add it for all the others. This feature allows teachers to effortlessly create new assignments, or students to practice by themselves with potentially infinitely many exercises.
- A new and **improved user interface** that allows teachers and students to navigate the increased number of problem types and assignments. Furthermore, each problem type comes with an intuitive user interface (e.g., for drawing pushdown automata).
- An improved **infrastructure** for the use in large courses, in particular, incorporating login systems (e.g. *LDAP* or *OAuth*), getting a certified mapping from users to students and enabling teachers to grade homework or exams.
- A **user study** run on a class of 950 students to assess the effectiveness and usability of Automata Tutor v3. In our survey, students report to have *learned quickly*, *felt confident*, and *enjoyed* using Automata Tutor v3, and found it *easy to use*. Most importantly, students found the feedback given by the tool to be *useful* and claimed they *understood more* after using the tool and felt *better prepared* for an upcoming exam. In our personal experience, the tool saves us dozens of thousands of corrections in each single course.

## Automata Tutor in a Nutshell

Automata Tutor is an online education tool created to support courses teaching basic concepts in automata and formal languages [7]. In this section, we describe how Automata Tutor helps teachers run large courses and students learn efficiently in such courses.

**Fig. 1.**
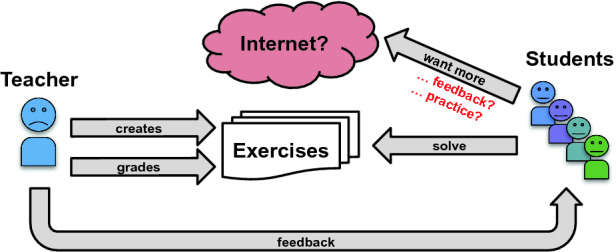
Common structure of practical sessions for CS classes.

*Learning Without Automata Tutor.* Figure 1 schematically shows a student-teacher interaction in a course taught without an online tutoring system. The teacher creates exercises, grades them manually, and (sometimes) manually provides personalized feedback to the students. This type of interaction has many limitations: (1) it is asynchronous (i.e., the student has to wait a long time for what is often little feedback) and does not scale to large classrooms, posing strenuous amount of work on teachers, (2) it does not guarantee consistency in the assigned grades and feedback, and (3) it does not allow students to revise their solutions upon receiving feedback as the teachers often release a solution to all students as part of the feedback and do not grade new submissions.

Another drawback of this interaction is the limited number of problems students can practice on. Because teachers do not have the resources to create many practice problems and provide feedback for them, students are often forced to search the Internet for old exams and practice sheets or even exercises from other universities. Due to the lack of feedback, this chaotic search for practice problems often ends up confusing the students rather than helping them.

**Fig. 2.**
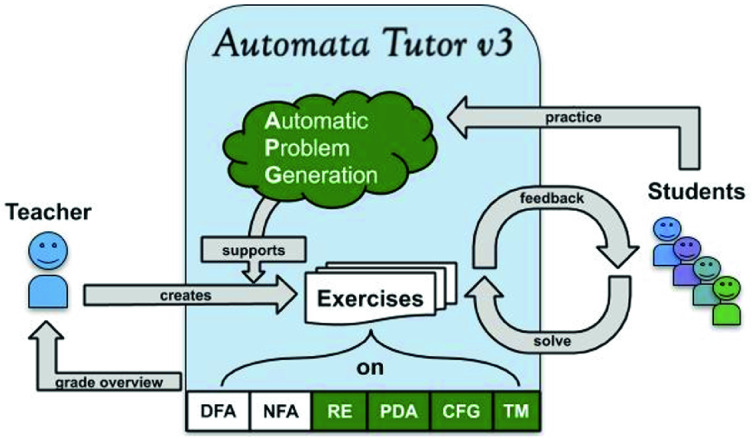
Overview of Automata Tutor v3 (our contributions in green). The teacher creates exercises on various topics. The students solve the exercises in a feedback cycle: After each attempt they are automatically graded and get personalized feedback. The teacher has access to the grade overview. For additional practice, students can generate an unlimited number of new exercises using the automatic problem generation. (Color figure online)

**Fig. 3.**
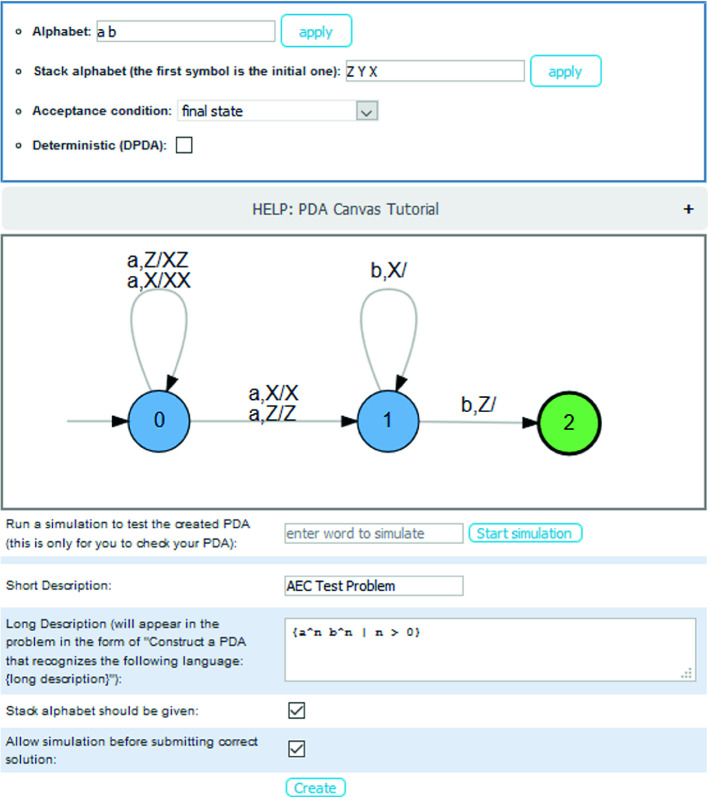
Creating a new problem of type “PDA Construction”.

**Fig. 4.**
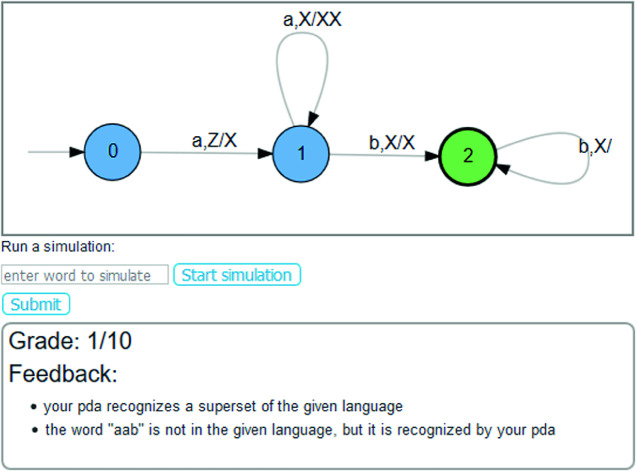
Feedback received when solving the problem created in Fig. 3.

*Learning with Automata Tutor.* Figure 2 shows the improved interaction offered by Automata Tutor v3. Here, a teacher creates the problem instances with the help of the tool. The problems are then posed to the students and, *no matter how large a class is*, Automata Tutor automatically grades the solution attempts of students right when they are submitted and immediately gives detailed and personalized feedback for each submission. If required, e.g. for a graded homework, it is possible to restrict the number of attempts. Using this feedback, the students can immediately try the problem again and learn from their mistakes. As shown in a large user study run on the first version of Automata Tutor [6], this fast feedback cycle is encouraging for students and results in students spontaneously exploring more practice problems and engaging with the course material. Additional practice is supported by the automatic problem generation, with the same level of detailed and personalized feedback as before without increasing the workload of the teacher. Furthermore, automatic problem generation can assist the teacher in creating new exercises. Finally, whenever necessary, the teacher can download an overview of all the grades.

*Improved User interface.* Automata Tutor is an online tool which runs in the most used browsers. A new collapsible navigation bar groups problems by topic, facilitating quick access to exercises and displaying the structure of the course (see Figure 6 in [5, Appendix B]). To create a new exercise, a teacher clicks the “+”-button and is presented the view of Fig. 3. In this case, the drawing canvas allows to easily specify the sample solution pushdown automaton. Similarly, when students solve this exercise, they draw their solution attempt also on the canvas. After submitting, they receive their personalized feedback and grade (see example in Fig. 4). For the automatic problem generation, a dropdown menu to select the problem type and a slider to select the difficulty is displayed together with the list of all problems the user has generated so far (see the screenshot in Figure 7 in [5, Appendix B]).

## Design

### University and Course Management

While Automata Tutor can be used for independent online practice, one of the main advantages is its infrastructure for large university courses. To this end, it is organized in *courses*. A course is created and supervised by one or more teachers. Together, they can create, test and edit exercises. The students cannot immediately see the problems, but only after the teachers have decided to pose them. This involves setting the maximum number of points, the number of allowed attempts as well as the start and end date.

To use Automata Tutor, students must have an account. One can either register by email or, in case the university supports it, login with an external login service like *LDAP* or *Oauth*. When using the login service of their university, teachers get a certified mapping from users to students and enabling teachers to use Automata Tutor v3 for grading homework or exams.

Students can enroll in a course using a password. Enrolled students see all posed problems and can solve them (using the allowed number of attempts). The final grade can be accessed by the teachers in the grade overview.

### New Problem Types

In this section, we list the problem types newly added to Automata Tutor v3. They are all part of the course [10] and a detailed description of each problem can be found in [5, Appendix A], including the basic theoretical concept, how a student can solve such a problem, what a teacher has to provide to create a problem, the idea of the grading algorithm, and what feedback the tool gives.

- *RE/CFG/PDA Words:* Finding words in or not in the language of a regular expression, context free grammar or pushdown automaton.
- *RE/CFG/PDA Construction:* Given a description of a language, construct a regular expression, context free grammar or pushdown automaton.
- *RE to NFA:* Given a regular expression, construct a nondeterministic-finite automaton.
- *Myhill-Nerode Equivalence Classes:* There are two subtypes: either, given a regular expression and two words, find out whether they are equivalent w.r.t. the language, or, given a regular expression and a word, find further words in the same equivalence class.
- *Pumping-Lemma Game:* Given a language, the student has to guess whether it is regular or not and then plays the game as one of the quantifiers.
- *Find Derivation:* Given a context free grammar and a word, the student has to specify a derivation of that word.
- *CNF:* Given a context free grammar, the student has to transform it into Chomsky Normal Form.
- *CYK:* Given a context free grammar in CNF and a word, the student has to decide whether the word is in the language of the grammar by using the Cocke–Younger–Kasami algorithm.
- *While to TM:* Given a while-program (a Turing-complete programming language with very restricted syntax), construct a (multi-tape) Turing machine with the same input-output behaviour.

### Automatic Problem Generation

Automatic Problem Generation (APG) allows one to generate new exercises of a requested *difficulty* level and problem type. This allows students to practice independently and supports teachers when creating new exercises. While APG is currently implemented for four CFG problem types and for the problem type “While to TM”, it can be easily extended to other problem types by providing the following components:

- **Procedure for generating exercises at random** either from given basic building blocks or from scratch.
- **A “quality” metric** $$ qual (E)$$ for assessing the quality of the generated exercise *E*, ranging from trivial or infeasible to realistic.
- **A “difficulty” metric** $$ diff (E)$$ for assessing the difficulty of *E*.

Given these components, Automata Tutor generates a new problem with a given minimum difficulty $d_{\min }$ and maximum difficulty $d_{\max }$ as follows. Firstly, 100 random exercises are generated. Secondly, Automata Tutor chooses exercises *E* with the best quality such that $d_{\min } \le diff (E) \le d_{\max }$.

Concretely, for the CFG problem types, CFGs with random productions are generated and sanitized. Resulting CFGs that do not accept any words or have too few productions are excluded using the quality metric. The difficulty metric always depends on the number of productions; additionally, depending on the exact problem type, further criteria are taken into account.

For the problem type “While to TM” we use an approach similar to the one suggested in existing tools for automatic problem generation [15, 18]: We handcrafted several *base programs* which are of different difficulty level. In the generation process, the syntax tree of such a base program is abstracted and certain modifying operations are executed; these change the program without affecting the difficulty too much. E.g. we choose different variables, switch the order of if-else branches or change arithmetic operators. Then several programs are generated and those of bad quality are filtered out. A program is of bad quality if its language is trivially small or if it contains infinite loops; since detecting these properties is undecidable, we employ heuristics such as checking that the loops terminate for all inputs up to a certain size with a certain timeout.

## Implementation and Scalability

Automata Tutor v3 is open source and it consists of a frontend, a backend, and a database. It also provides a developer’s manual for creating new exercises.

The frontend, written in scala, renders the webpage. The drawing canvases for the different automata and the Turing machines rely on javascript. The frontend and backend communicate using XML objects.

The backend, written in C#, contains methods to unpack the xml of the frontend to compute the grade and feedback for solutions. It is also used to check the syntax of exercises and for the automatic problem generation. It relies on AutomataDotNet^2^, a library that provides efficient algorithms for automata and regular expressions.

The database keeps track of existing users, problems and courses. It uses the H2 Database Engine.

All the new parts of Automata Tutor v3 were developed and tested over the last 3 years at TU Munich, where they were used to support the introductory theoretical computer science course. This local deployment served as an important test-bed before publicly deploying the tool online at large scale. Due to its modular structure, the tool is easily scalable by having multiple frontends and backends together with a load distributor. This approach has successfully scaled to 950 concurrent student users; for this, we used 7 virtual machines: 3 hosting frontends, 3 hosting backends (each with 2 cores 2.60 GHz Intel(R) Xeon(R) CPU and 4 GB RAM), and 1 for load distribution and the database (with 4 such cores and 8 GB RAM). We will scale the number of machines based on need.

## Evaluation and User Study

**Large-Class Deployment.**In the latest iteration of the TU Munich course in 2019, we used Automata Tutor v3 (in the following denoted as AT) in a mandatory homework system for a course with about 950 students; the homework system also included written and programming exercises. In total, we posed 79 problems consisting of 18 homework and 61 practice problems. The teachers saved themselves the effort of correcting 26,535 homework exercises, and the students used AT to get personalized feedback for their work 76,507 times. On average, each student who used AT did so 107 times.

**Student Survey Results.**At the end of the course, we conducted an anonymized survey, based on the System Usability Survey [4]. 14.6% of the students in the course answered the survey, which is an ordinary rate of return for an online questionnaire, especially given that there was no incentive. The students were given statements to judge on a Likert scale from 1 to 5 (strongly disagree to strongly agree). We define “The students agreed with the following statement” to mean that the average and median scores were at least 4 and less than 10% of the students chose a score below 3. Dually, if the students disagreed with the statement with median and average score that was at most 2 and less than 10% having a score greater than 3, we say that they “agreed with the negation of the statement”. For all statements that do not satisfy either of the criteria, we report mixed answers. The full survey results can be found in [5, Appendix C].

*Usability.* Regarding the usability of the tool, the students agreed with the following statements:

- I quickly learned to use the AT.
- I do *not* need assistance to use the AT.
- I feel confident using the AT.
- The AT is easy to use.
- I enjoy using the AT/the AT is fun to use.

However, there were lots of valuable suggestions for improvements, many of which we have implemented since then. Moreover, the survey also revealed space for improvement, in particular for streamlining as documented by the following statements where the answers were more mixed:

- The AT is unnecessarily complex.
- The canvas for drawing is intuitive.
- The use of AT is self-explanatory.

*Usefulness.* Regarding how useful AT was for learning, the students agreed with the following statements:

- I understand more after using the AT.
- I prefer using the AT to using pen and paper exercises (12.9% disagreed, but median and average are 4).
- The feedback of the AT was helpful and instructive.
- The exercises within the AT are well-designed.
- The AT fits in well with the programming tasks and written homework.
- The AT did *not* hinder my learning.
- I feel better prepared for the exam after using AT.
- The feedback of the AT was *not* misleading/confusing.

**Fig. 5.**
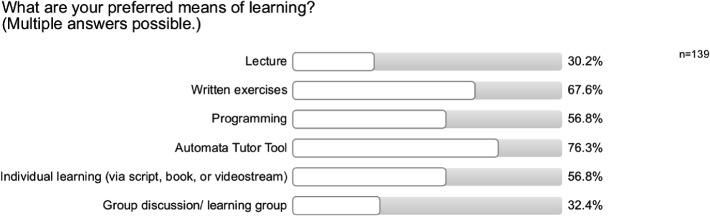
Question from the survey we conducted to evaluate Automata Tutor, showing that the tool is preferred by a majority of students.

Note that there are no statements with mixed or negative answers regarding the usefulness. Additionally, as shown in Fig. 5, when we asked students about their preferred means of learning, AT gets the highest approval rate, being preferred to written or programming exercises as well as lectures.

Overall, this class deployment of Automata Tutor v3 and the accompanying surveys were great successes, and showed how the tool is of extreme value for both students and teachers, in particular for such large a course.

## Conclusion

This paper presents the third version of Automata Tutor, an online tool helping teachers and students in large automata/computation theory courses. Automata Tutor v3 now supports automated grading and feedback generation for a wide variety of problems and, for some of them, even automatic generation of new problem instances. Furthermore, it is easy to extend and we invite the community to contribute by implementing further exercises. Finally, our experience shows that Automata Tutor v3 improves the economical aspects of teaching greatly as it scales effortlessly with the number of students.

Earlier versions of Automata Tutor have already been adopted by thousands of students at dozens of schools and we hope this paper allows Automata Tutor v3 to help even more students and teachers around the world.

## Footnotes

1 https://automata.model.in.tum.de.

2 https://github.com/AutomataDotNet/Automata.

## References

[1] Gradiance online accelerated learning. http://www.newgradiance.com/

[2] Ahmed, U.Z., Gulwani, S., Karkare, A.: Automatically generating problems and solutions for natural deduction. In: IJCAI 2013, Proceedings of the 23rd International Joint Conference on Artificial Intelligence, 3–9 August 2013, Beijing, China (2013)

[3] Alur, R., D’Antoni, L., Gulwani, S., Kini, D., Viswanathan, M.: Automated grading of DFA constructions. In: Proceedings of the Twenty-Third International Joint Conference on Artificial Intelligence, IJCAI 2013, pp. 1976–1982. AAAI Press (2013)

[4] Brooke J, Jordan PW, Thomas B, McClelland IL, Weerdmeester B (1996). Sus-a quick and dirty usability scale. Usability Evaluation in Industry.

[5] D’Antoni, L., Helfrich, M., Kretinsky, J., Ramneantu, E., Weininger, M.: Automata tutor v3. CoRR, abs/2005.01419 (2020)

[6] D’antoni, L., Kini, D., Alur, R., Gulwani, S., Viswanathan, M., Hartmann, B.: How can automatic feedback help students construct automata? ACM Trans. Comput. Hum. Interact. **22**(2), 1–24 (2015)

[7] D’ Antoni, L., Weavery, M., Weinert, A., Alur, R.: Automata tutor and what we learned from building an online teaching tool. Bull. EATCS, **3**(117), 144–158 (2015)

[8] National Research Council: How People Learn: Brain, Mind, Experience, and School: Expanded Edition. The National Academies Press, Washington, D.C (2000)

[9] Gulwani S, Korthikanti VA, Tiwari A (2011). Synthesizing geometry constructions. SIGPLAN Not..

[10] Hopcroft JE, Motwani R, Ullman JD (2007). Introduction to Automata Theory, Languages, and Computation.

[11] Itzhaky S, Gulwani S, Immerman N, Sagiv M, McMillan K, Middeldorp A, Voronkov A (2013). Solving geometry problems using a combination of symbolic and numerical reasoning. Logic for Programming, Artificial Intelligence, and Reasoning.

[12] Patterson, D.: Why are English majors studying computer science? November 2013

[13] Rahn, M., Waldmann, J.: The leipzig autotool system for grading student homework. Functional and Declarative Programming in Education (FDPE) (2002)

[14] Shekhar, V.S., Agarwalla, A., Agarwal, A., Nitish, B., Kumar, V.: Enhancing JFLAP with automata construction problems and automated feedback. In: Parashar, M., et al. (ed.) Seventh International Conference on Contemporary Computing, IC3 2014, Noida, India, 7–9 August 2014, pp. 19–23. IEEE Computer Society (2014)

[15] Shenoy, V., Aparanji, U., Sripradha, K., Kumar, V.: Generating DFA construction problems automatically. In: 2016 International Conference on Learning and Teaching in Computing and Engineering, LaTICE, pp. 32–37. IEEE (2016)

[16] Singh, R., Gulwani, S., Rajamani, S.K.: Automatically generating algebra problems. In: Proceedings of the Twenty-Sixth AAAI Conference on Artificial Intelligence, 22–26 July 2012 Toronto, Ontario, Canada (2012)

[17] Singh, R., Gulwani, S., Solar-Lezama, A.: Automated feedback generation for introductory programming assignments. In: Proceedings of PLDI 2013, New York, NY, USA, pp. 15–26. ACM (2013)

[18] Weinert, A.: Problem generation for DFA construction (2014). https://alexanderweinert.net/papers/2014dfageneration.pdf. Accessed 04 May 2020

